# Impact of the Preoperative Controlling Nutritional Status (CONUT) Score on the Survival after Curative Surgery for Colorectal Cancer

**DOI:** 10.1371/journal.pone.0132488

**Published:** 2015-07-06

**Authors:** Yasuhito Iseki, Masatsune Shibutani, Kiyoshi Maeda, Hisashi Nagahara, Hiroshi Ohtani, Kenji Sugano, Tetsuro Ikeya, Kazuya Muguruma, Hiroaki Tanaka, Takahiro Toyokawa, Katsunobu Sakurai, Kosei Hirakawa

**Affiliations:** Department of Surgical Oncology, Osaka City University Graduate School of Medicine, Osaka, Japan; School of Medicine, Fu Jen Catholic University, TAIWAN

## Abstract

**Background:**

Recently, the preoperative immune-nutritional status has been reported to correlate with the survival rate in patients with colorectal cancer (CRC). However, there have been no reports on the relationship between the controlling nutritional status (CONUT) score and the clinical outcome after curative surgery for CRC. We herein evaluated the prognostic significance of the CONUT score in patients with CRC, and then compared the accuracy of the CONUT score and the prognostic nutritional index (PNI) as a predictor of survival.

**Methods:**

We retrospectively reviewed a database of 204 patients who underwent curative surgery for Stage II/III CRC. Patients were divided into two groups according to the CONUT score and the PNI.

**Results:**

The five-year cancer-specific survival (CSS) rate was significantly higher at 92.7% in the low CONUT group, compared to a rate of 81.0% in the high CONUT group (p=0.0016). The five-year CSS was 71.2% in the low PNI group and 92.3% in the high PNI group, which showed a significant difference (p=0.0155). A multivariate analysis showed that lymph node metastasis and the CONUT score were independent risk factors for CSS.

**Conclusion:**

This study suggested that the CONUT score is a strong independent predictor of the survival among CRC patients.

## Introduction

Colorectal cancer (CRC) is the third most common cancer in the world [1].

Although the surgical procedures and chemotherapy for CRC have improved, the clinical outcome of CRC is still poor, as one-third of the patients who undergo curative resection die within five years after surgery [2]. Therefore, it is necessary to identify biomarkers that can predict the prognosis and individualize the therapy based on the stratification of risks. Many studies about the potential prognostic factors for CRC have been carried out, and the preoperative immune-nutritional status has been reported to correlate with the survival for CRC [3–7].

Recently, the prognostic nutritional index (PNI), which was calculated from the serum albumin concentration and the total peripheral lymphocyte count, has been used to predict the risk of postoperative complications [8], and it has also been reported to correlate with the survival in CRC patients [3, 9]. Regarding the PNI, we consider that the serum albumin concentration tends to be excessively emphasized. However, the serum albumin concentration has been reported to be easily influenced by not only the nutritional status, but also by changes in the body fluid volume, such as those due to the dehydration/fluid retention status and inflammation caused by chronic disease [10, 11]. Therefore, this study focused on evaluating the Controlling Nutritional Status (CONUT) score [10].

The CONUT score is an index calculated from the following factors; the serum albumin concentration, the total peripheral lymphocyte count and total cholesterol concentration. Total cholesterol concentration has also been reported to correlate with the progression of cancer [11]. A more accurate evaluation can be obtain by reducing the importance of the serum albumin concentration and adding the total cholesterol concentration to the evaluation criteria [12]. Although the prognostic significance of the PNI has been reported in numerous previous reports, there have been no reports on the relationship between the CONUT score and the clinical outcome after curative surgery for CRC.

The aim of this retrospective study is to determine whether the preoperative CONUT score could be a useful predictor of the survival in patients with CRC, and to compare the accuracy of the CONUT score and the PNI as a predictor of the survival rate of such patients.

## Patients and Methods

### Patients

We retrospectively reviewed a database of 204 patients who underwent curative surgery for Stage II/III CRC at the Department of Surgical Oncology, Osaka City University, Japan between April 2004 and December 2009. We performed a retrospective review of 204 patients with Stage II or III CRC. We obtained written informed consent from the patients for participation and the study protocol was approved by the ethics committee of Osaka City University. Our investigation was conducted according to the principles expressed in the Declaration of Helsinki. The resected specimens were assessed using The International Union Against Cancer (UICC) staging classification of colorectal cancer [13]. All patients were followed up until April 2012 or until their deaths.

The indications for undergoing adjuvant chemotherapy included patients with Stage III or high-risk Stage II disease. T4 tumors, lymphatic vessel invasion, blood vessel invasion, high-grade histology, presentation with obstruction/perforation and inadequate lymph node sampling were defined as high-risk Stage II disease. The decision of whether or not the patients should undergo adjuvant chemotherapy was determined by the surgeons with the patients’ consent. The doctors judged the indication for chemotherapy, for example, the criteria, performance status, general condition, age and patient’s wishes.

There were no patients treated with neoadjuvant chemotherapy in this study.

### Methods

The preoperative blood samples were obtained within two weeks before the operation. The CONUT score was calculated using the serum albumin concentration, peripheral lymphocyte count and the total cholesterol concentration, as described in Table 1 [12].

**Table 1:** Assessment of the nutritional status using the CONUT score.

	None	Light	Moderate	Severe
Serum albumin (g/dL)	≥3.50	3.00–3.49	2.50–2.99	<2.50
Score	0	2	4	6
Total lymphocyte count (/mm^3^)	≥1600	1200–1599	800–1199	<800
Score	0	1	2	3
Total cholesterol (mg/dL)	≥180	140–179	100–139	<100
Score	0	1	2	3
Add scores	≤2 Low CONUT group			
	3≤ High CONUT group			

CONUT: controlling nutritional status; PNI: prognostic nutritional index; SD: Standard deviation de Ulibarri Perez JI, et al. (2005) Nutr Hosp.[10]

**Fig 1:**
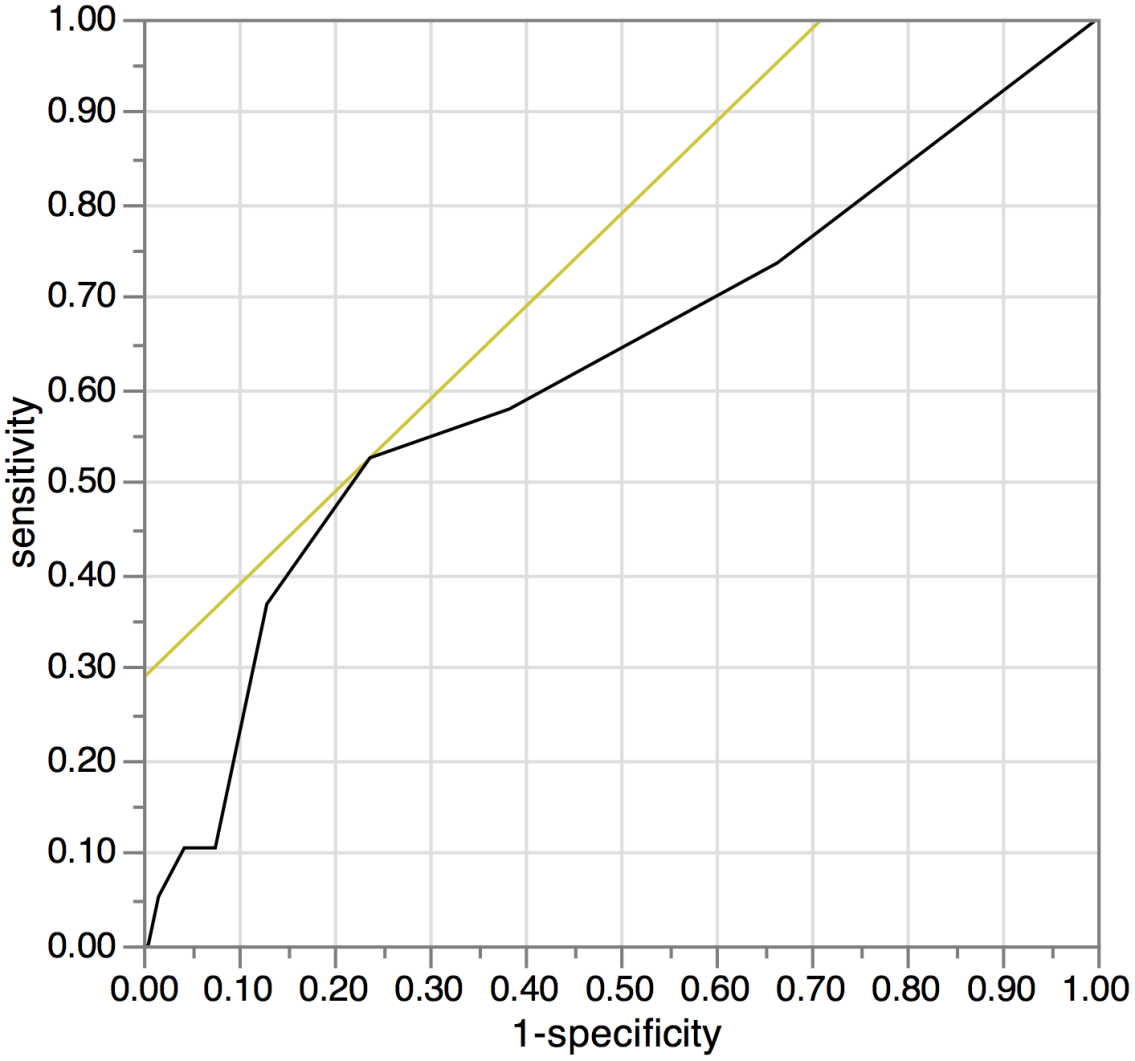
The receiver operating characteristic (ROC) curve for the controlling nutritional status (CONUT). We used the continuous variable Controlling Nutritional Status (CONUT) as the test variable and 5-year cancer-specific survival as the state variable. An investigation of the cut-off value for the CONUT score using the receiver operating characteristic (ROC) curve showed the most appropriate cut-off value for the CONUT score to be 3 (AUC; 0.624, 95%CI: 0.476–0.771, p = 0.076, the sensitivity was 0.5263 and the specificity was 0.7622.). We indicated the ROC curve on Fig 1. Therefore, we set 3 as the cut-off value for the CONUT score in this study and classified the patients into high CONUT (≥3) and low CONUT (≤2) groups.

We used the continuous variable Controlling Nutritional Status (CONUT) as the test variable and cancer-specific survival as the state variable. An investigation of the cut-off value for the CONUT score using the receiver operating characteristic (ROC) curve showed the most appropriate cut-off value for the CONUT score to be 3 (AUC; 0.624, 95%CI: 0.476–0.771, p = 0.076, the sensitivity was 0.5263 and the specificity was 0.7622.). We indicated the ROC curve on Fig 1. Therefore, we set 3 as the cut-off value for the CONUT score in this study and classified the patients into high CONUT (≥3) and low CONUT (≤2) groups.

The following formula was used to calculate PNI: 10 × serum albumin concentration (g/dL) + 0.005 × total peripheral lymphocyte count (per mm^3^) [8]. As with previous reports in which a low PNI (<40) was reported to be a prognostic factor for CRC [3], we also set 40 as the cut-off value of the PNI in the present study. The patients were divided into two groups; a low PNI (<40) group and a high PNI (≥ 40) group.

Regarding the relapse-free survival, relapse was noted as cancer relapse and deaths of all causes was treated as relapse. The survival times were measured from the date of the operation to the date of being lost to follow-up, the date of relapse, the date of death of all causes or April 30, 2012, whichever occurred first.

As to cancer-specific survival, deaths noted to be caused by colorectal cancer were treated as deaths, and other deaths were regarded as censored events. The survival times were measured from the date of operation to the date of being lost to follow-up, the date of death or April 30, 2012, whichever occurred first.

Differences between the groups were analyzed using the χ^2^ test and the Wilcoxon signed rank test. The duration of survival was calculated according to the Kaplan-Mayer method. Differences in the survival curves were assessed with the log-rank test. A multivariate analysis of the clinicopathological factors for survival was performed using a Cox proportional hazard model. Statistical significance was set at a value of p<0.05. The JMP 11 software program (SAS Institute, Cary, NC, USA) was used to analyze the data.

## Results

### Clinical characteristics

The patient characteristics are shown in Table 2. Fifty-four patients were classified into the high CONUT group and one hundred and fifty patients were classified into the low CONUT group, based on the cut-off value of 3. One hundred and seventy-seven patients were classified into the high PNI group and twenty-seven patients were classified into the low PNI group, based on the cut-off value of 40. All patients with a low PNI were included in the high CONUT group (Table 3).

**Table 2:** The relationships between the CONUT score and PNI and the clinical background of the patients.

	The CONUT score			PNI		
	High (N = 54)	Low(N = 150)	p-value	≥40 (N = 177)	<40 (N = 27)	p-value
Sex						
Male	27	85	0.7110	101	11	0.1131
Female	27	65		76	16	
Age (years)						
mean ± SD	66.09±9.23	71.13±11.57	0.0001	66.37±9.99	74.33±8.15	0.0001
Tumor location						
Colon	41	87	0.0169	106	22	0.0224
Rectum	13	63		71	5	
Tumor size (cm)						
mean ± SD	5.05±0.26	4.38±0.15	0.1068	4.47±1.80	5.09±2.35	0.2611
Depth of tumor invasion						
T1,2,3	32	105	0.1539	120	17	0.6213
T4	22	45		57	10	
Lymph node metastasis						
Negative	30	89	0.6298	104	15	0.7539
Positive	24	61		73	12	
Lymphatic vessel invasion						
Negative	14	40	0.9266	46	8	0.6834
Positive	38	105		125	18	
Venous invasion						
Negative	44	116	0.4573	138	22	0.6267
Positive	8	29		33	4	
Adjuvant chemotherapy						
No	31	66	0.0907	77	20	0.0030
Yes	23	84		100	7	
Complications						
No	44	120	0.8141	145	19	0.1591
Yes	10	30		32	8	

CONUT: controlling nutritional status; PNI: prognostic nutritional index; SD: Standard deviation

**Table 3:** The distribution according to the CONUT score/PNI and the five-year survival rate.

		The CONUT score	
		Low	High
PNI	Low	0	27 (71.2%)
	High	150 (92.7%)	27 (89.1%)

Number (five-year cancer-specific survival rate, %), (p<0.0001)

CONUT: controlling nutritional status; PNI: prognostic nutritional index

There were no operation-related deaths or hospitalization deaths in this study. Regarding complications, the number of cases with more than Clavien-Dindo classification 2 complications was 40 (19.6%). Specifically, 10 patients had anastomotic leakage, 23 patients had infectious complications and 19 patients had other complications.

Ninety-seven of 160 patients (60.8%) received adjuvant chemotherapy. As a result, 93 patients were given the 5-fluolouracil (FU) regimen and four patients were given the 5-FU+Oxaliplatin regimen.

The primary tumor was resected in all patients. In cases of recurrent disease, some tumors were removed surgically, and one patient chose chemotherapy. A total of 24 patients (51.1%) received resection for recurrent tumors out of 47 patients with recurrent disease.

### The correlations between the CONUT/PNI and the clinicopathological factors

The CONUT score had a significant relationship with the age (p = 0.0016) and tumor location (p = 0.0168). The PNI had significant relationship with the age (p = 0.0001), tumor location (p = 0.0224), and adjuvant chemotherapy (p = 0.0030) (Table 2).

### Survival analysis according to the CONUT and PNI

**Fig 2:**
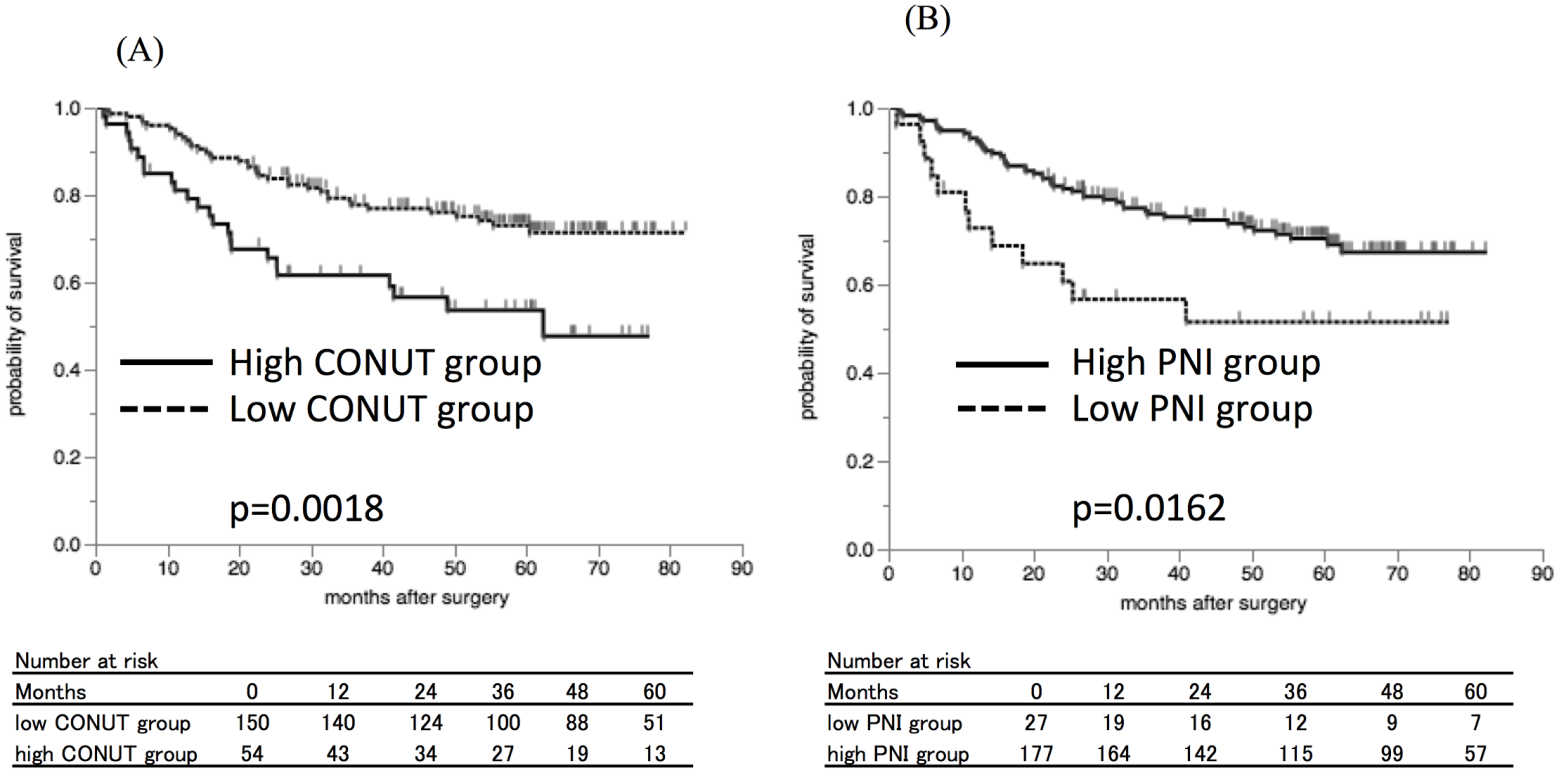
The Kaplan-Mayer survival curves for the relapse-free survival (RFS). A) The survival curves according to the CONUT score. The relapse-free survival rates were significantly worse in the high CONUT group compared to the low CONUT group (p = 0.0018). B) The survival curves according to the PNI. The relapse-free survival rates were significantly worse in the low PNI group compared to the high PNI group (p = 0.0162).

The five-year relapse-free survival (RFS) rate was 73.0% in the low CONUT group and 53.6% in the high CONUT group (Fig 2), with a significant difference between the groups (p = 0.0018). In addition, the five-year RFS was significantly lower at 51.5% in the low PNI group compared to 70.4% in the high PNI group (Fig 2, p = 0.0162).

**Fig 3:**
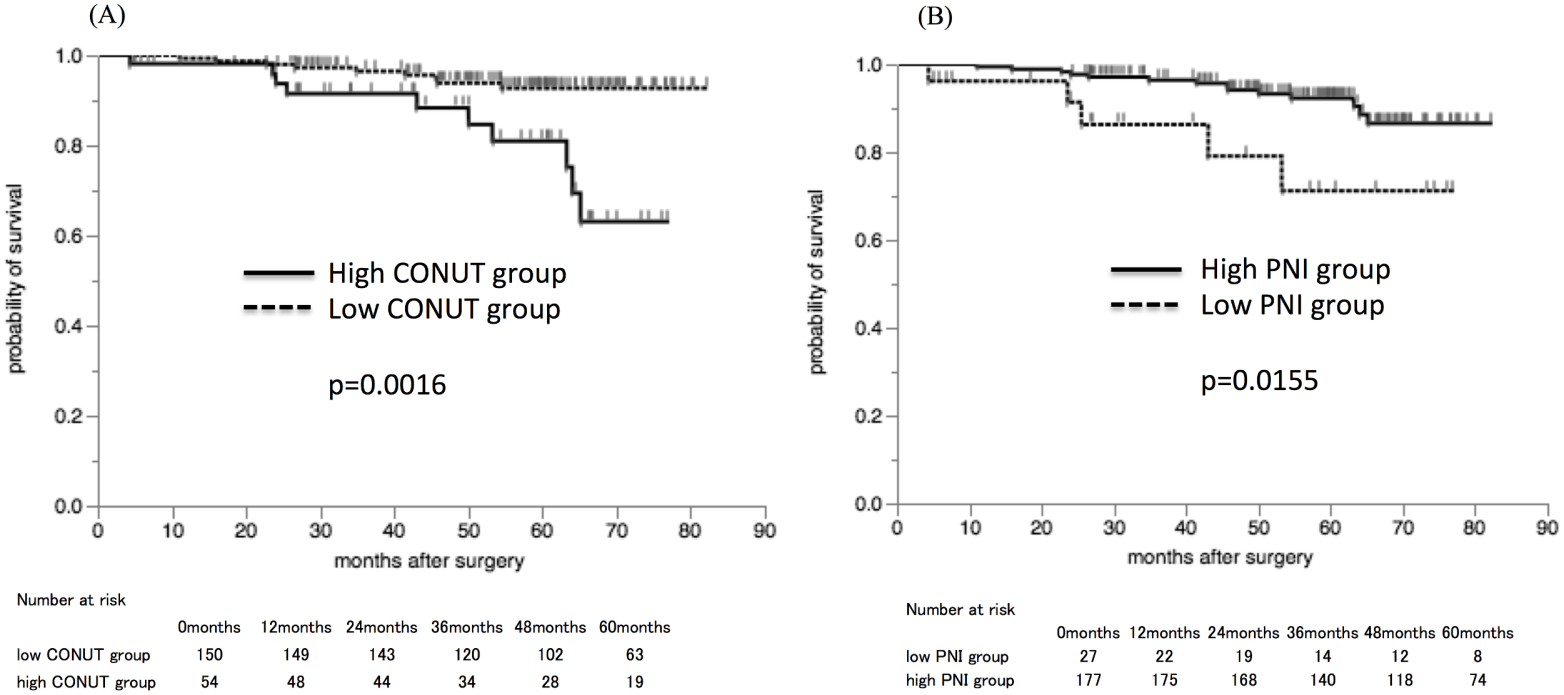
The Kaplan-Mayer survival curves for the cancer-specific survival (CSS). A) The survival curves according to the controlling nutritional status (CONUT) score. The cancer-specific survival rates were significantly worse in the high CONUT group compared to the low CONUT group (p = 0.0016). B) The survival curves according to the prognostic nutritional index (PNI). The cancer-specific survival rates were significantly worse in the low PNI group compared to the high PNI group (p = 0.0155).

The five-year cancer-specific survival (CSS) was 92.7% in the low CONUT group and 81.0% in the high CONUT group (Fig 3), and there was a significant difference between the low and high CONUT groups (p = 0.0016). Moreover, the five-year CSS was significantly lower at 71.2% in the low PNI group compared to 92.7% in the high PNI group (Fig 3, p = 0.0155).

### Prognostic factors influencing the RFS and the CSS

The correlations between the RFS and clinicopathological factors are shown in Table 4. The patient sex, age, lymphatic vessel invasion, vessel invasion, lymph node metastasis, preoperative carbohydrate antigen 19–9 (CA19-9 level), CONUT score and PNI were significantly associated with the RFS. When a multivariate analysis was performed, T4 tumor, preoperative carcinoembryonic antigen (CEA) level and adjuvant chemotherapy, which are known as prognostic factors well [3, 9], were added as covariates. A multivariate analysis showed that sex (Odds ratio (OR) = 2.135, 95% confidence interval(CI); 1.177–4.036, p = 0.0121), age (OR = 1.864, 95%CI; 1.009–3.482, p = 0.0469), venous invasion(OR = 2.069, 95%CI; 1.051–3.955, p = 0.0359) and the preoperative CA19-9 level (OR = 2.816, 95%CI; 1.252–5.997, p = 0.0134) were independently associated with the RFS (Table 4).

**Table 4:** The results of the univariate and multivariate analyses of the prognostic factors for the relapse-free survival (RFS).

	Univariate			Multivariate		
	OR	95%CI	p-value	OR	95%CI	p-value
Sex (male)	1.718	1.027–3.960	0.0391	2.135	1.177–4.036	0.0121
Age (≥70)	2.193	1.326–3.689	0.0022	1.864	1.009–3.482	0.0469
Tumor locatiom (rectum)	1.778	0.851–3.963	0.1280			
Tumor size (≥4.0cm)	1.019	0.600–1.692	0.9415			
Depth of tumor invasion (T4)	1.479	0.885–2.440	0.1335	1.548	0.812–3.020	0.1861
Lymphatic vessel invasion (positive)	2.085	1.102–4.369	0.0225	1.838	0.832–4.643	0.1379
Venous invasion (positive)	2.332	1.320–3.974	0.0043	2.069	1.051–3.955	0.0359
Lymph node metastasis (positive)	2.167	1.313–3.623	0.0025	1.783	0.929–3.470	0.0822
Preoperative CEA (>5ng/ml)	1.268	0.762–2.097	0.3576	1.137	0.628–2.117	0.6750
PreoperativeCA19-9 (>37U/ml)	3.080	1.554–5.645	0.0021	2.816	1.252–5.997	0.0134
Adjuvant chemotherapy (No)	1.277	0.774–2.137	0.3394	1.002	0.508–2.013	0.9952
The CONUT score (≥3)	2.210	1.307–3.662	0.0036	1.836	0.844–3.713	0.1206
PNI (<40)	2.130	1.082–3.867	0.0301	1.011	0.384–2.600	0.9825

OR: odds ratio; CI: confidence interval; CEA: carcinoembryonic antigen; CA19-9: carbohydrate antigen 19–9; CONUT: controlling Nutritional status; PNI: prognostic nutritional index

The correlations between the CSS and the clinicopathological factors are shown in Table 5. A univariate analysis indicated that lymph node metastasis, the preoperative CA19-9 level, the CONUT score and the PNI were significantly associated with the CSS. When a multivariate analysis was performed, T4 tumor, preoperative carcinoembryonic antigen (CEA) level and adjuvant chemotherapy were added as covariates. A multivariate analysis showed that only lymph node metastasis (OR = 3.680, 95%CI; 1.106–14.914, p = 0.0330) and the CONUT score (OR = 4.212, 95%CI; 1.215–13.350, p = 0.0251) were independently associated with the CSS (Table 5).

**Table 5:** The results of the univariate and multivariate analyses of the prognostic factors for the cancer-specific survival (CSS).

	univarate			multivariate		
	OR	95%CI	p-value	OR	95%CI	p-value
Sex(male)	1.297	0.521–3.489	0.5820			
Age (≥70)	2.435	0.977–6.560	0.0563			
Tumor location (rectum)	1.081	0.324–3.764	0.8982			
Tumor size (≥4.0cm)	0.783	0.311–1.944	0.5950			
Depth of tumor invasion (T4)	1.325	0.513–3.275	0.5487	1.140	0.409–3.279	0.8017
Lymphatic vessel invasion (positive)	2.067	0.688–8.890	0.2118			
Venous invasion (positive)	1.640	0.530–4.290	0.3630			
Lymph node metastasis (positive)	4.390	1.677–13.601	0.0022	3.680	1.106–14.914	0.0330
Preoperative CEA (>5ng/ml)	1.514	0.591–3.879	0.3807	0.934	0.319–2.928	0.9027
Preoperative CA19-9 (>37U/ml)	3.734	1.197–9.910	0.0256	2.405	0.670–7.614	0.1687
Adjuvant chemotherapy (No)	2.417	0.924–7.487	0.0733	2.208	0.645–8.972	0.2139
The CONUT score (≥3)	3.839	1.546–9.673	0.0043	4.212	1.215–13.350	0.0251
PNI (<40)	3.300	1.063–8.634	0.0400	1.119	0.271–4.330	0.8700

OR: odds ratio; CI: confidence interval; CEA: carcinoembryonic antigen; CA19-9: carbohydrate antigen 19–9; CONUT: controlling Nutritional Status; PNI: prognostic nutritional index

### The comparison between the CONUT score and factors that comprise the CONUT score

The influence of the factors that comprise the CONUT score (albumin level, the total cholesterol level and the total peripheral lymphocyte count) on survival was examined.

The cut-off values for each of the factors were determined by their respective ROC curves on cancer-specific survival. The cut-off value for the albumin level was 3.5 g/dL, that for the total cholesterol level was 168 mg/dL and that for the total peripheral lymphocyte counts was 1170/mm^3^. In the univariate analysis for RFS, the albumin level (p = 0.025) and the total cholesterol level (p = 0.032) were found to be predictive factors (Table 6). In the univariate analysis for CSS, the albumin level and the total peripheral lymphocyte level were found to be predictive factors.

**Table 6:** The results of univariate analysis of the albmin, total cholesterol level and total peripheral lymphocytes count for the survival.

	Relapse free survival			Cancer specific survival		
	OR	95%CI	p-value	OR	95%CI	p-value
Albumin (<3.5g/dL)	2.013	1.105–3.481	0.0235	2.889	1.013–7.300	0.0475
Total cholesterol level (<168mg/dL)	1.783	1.049–2.963	0.0332	2.339	0.903–5.798	0.0784
Total peripheral lymphocyte count (<1170mm^3^)	1.431	0.744–2.558	0.2681	4.003	1.548–9.905	0.0054

OR: odds ratio; CI: confidence interval; CONUT: controlling Nutritional Status

The multivariate analysis for RFS indicated the CONUT score to be a more useful factor than the total cholesterol level and total peripheral lymphocyte counts (Tables 7, 8 and 9). The multivariate analysis showed the CONUT score to be superior to the serum albumin level and the total cholesterol score for predicting CSS. This study suggested that the CONUT score is a more useful factor for predicting survival than the individual factors that comprise the CONUT score (Tables 7, 8 and 9).

**Table 7:** The results of the multivariate analysis of the association between the CONUT score and the albumin level with relapse-free survival and cancer-specific survival.

	Relapse-free survival			Cancer-specific survival		
	OR	95%CI	p-value	OR	95%CI	p-value
CONUT (≥3)	2.04	0.962–3.989	0.0621	3.637	1.071–10.915	0.0393
Albumin (<3.5)	1.141	0.524–2.555	0.741	1.102	0.322–3.945	0.8764

OR: odds ratio; CI: Confidence interval.

**Table 8:** The results of the multivariate analysis of the association between the CONUT score and the total cholesterol level with relapse-free survival and cancer-specific survival.

	Relapse-free survival			Cancer-specific survival		
	OR	95%CI	p-value	OR	95%CI	p-value
CONUT (≥3)	1.966	1.127–3.363	0.0178	3.327	1.242–8.952	0.8017
Total cholesterol level (<168mg/dL)	1.440	0.821–2.474	0.1996	1.488	0.539–3.977	0.4340

OR: odds ratio; CI: confidence interval: CONUT: controlling nutritional status.

**Table 9:** The results of the multivariate analysis of the association between the CONUT score and the total peripheral lymphocyte count with relapse-free survival and the cancer-specific survival.

	Relapse-free survival			Cancer-specific survival		
	OR	95%CI	p-value	OR	95%CI	p-value
CONUT (≥3)	2.374	1.289–4.219	0.0062	2.513	0.825–7.449	0.104
Total peripheral lymphocyte count (<1170)	0.848	0.407–1.689	0.6452	2.332	0.767–7.106	0.1343

OR: odds ratio; CI: confidence interval; CONUT: controlling nutritional status

## Discussion

Various methods to evaluate the immune-nutritional status have been advocated, and recently the PNI has been reported to be associated with the postoperative survival in CRC patients [3–7, 14, 15]. Besides the indicating the nutritional status of a patient [16], the subjective global assessment (SGA) was also reported to be associated with the survival for CRC patients [4]. However, the SGA includes many subjective factors which require expert knowledge to accurately measure them [4], thereby limiting its clinical application.

The CONUT score, which was reported to correlate with the SGA, was developed to evaluate the nutritional status more easily and more objectively [12]. However, there have been no previous reports on the relationship between the preoperative immune-nutritional status and the survival after curative surgery for CRC using the CONUT score. This is therefore the first report to evaluate the prognostic significance of the CONUT score in patients with CRC.

The serum albumin concentration is influenced by not only the nutritional status [11], but also by many other factors, such as damage to hepatocytes, infection, inflammation, dehydration or fluid retention status, etc. [10, 17]. The lower albumin level in some patients may due to the production of cytokines and CRP, which modulate the production of albumin [11, 18]. It was shown that a systemic and chronic inflammatory response to CRC was associated with a reduction in the survival of CRC patients [19, 20]. It was also reported that as the inflammation due to cancer increased, the serum albumin concentration of the patients decreased. The poorer prognosis of the CRC patients with lower albumin concentration was associated with the presence of chronic and systemic inflammation [9].

The total peripheral lymphocyte count is one of the indicators of the immunological status [21]. T-lymphocytes play an important role in the immune response to cancer [22, 23]. Menges et al. revealed that lymphopenia caused by the systemic inflammatory response is characterized by significant depression of the innate cellular immunity, indicated by a marked decrease in T-4 helper lymphocytes and natural killer cells [24]. A decrease in T-lymphocytes was reported to correlate with a poor prognosis because of the inadequate immune response to cancer [22, 23]. As above, a decrease in the peripheral lymphocyte count is a poor prognostic factor in CRC patients [25].

Low serum cholesterol levels were reported to be associated with a poorer prognosis in patients with various cancers [11, 26, 27]. Although it remains unclear why a low serum cholesterol level is associated with a poor prognosis, hypocholesterolemia is not considered to be a cause of cancer, but to be induced by cancer [11]. There is increased expression of LDL receptor mRNA in tumor tissue than in normal tissue. The expression of LDL receptors on tumor cells makes them take up many LDLs [28], which decreases the serum cholesterol level [28]. In addition, the LDL cholesterol taken up into tumor cells increases tumor growth [29–31]. This hypothetical mechanism is supported by the reports that the serum cholesterol level increased after tumor resection [28]. A decrease in the serum cholesterol level means not only a lack of caloric intake, but also a loss of cholesterol from the cell membrane [10]. Hypocholesterolemia influences the cell membrane fluidity, which affects the mobility of cell surface receptors and their ability to transmit transmembrane signals [32]. Therefore, even if there are a sufficient number of immunocompetent cells present, they are unable to exert their immunological function against cancer cells due to the changes in their membranes [32, 33]. It has been hypothesized that this is why hypocholesterolemia is associated with a poor prognosis.

The PNI, which is the immune-nutritional index calculated using the serum albumin level and the peripheral lymphocyte count, has previously been reported to be associated with the survival in CRC patients [3, 9].

In the present report, the CONUT score more accurately predicted the survival in CRC patients than the PNI. Although the CONUT score and the PNI have common factors, they led to different results. Therefore, we examined the reasons why the CONUT score was superior to the PNI in predicting the prognosis. We found that all of the patients with a low PNI were included in the high CONUT group (Table 3). Because the CONUT score could detect the patients who were expected to have a poor prognosis, including some who were not detected by the PNI, the CONUT score was a more accurate prognostic indicator than the PNI. The patients with a high CONUT score who were not included in the low-PNI group had a low peripheral lymphocyte count and/or hypocholesterolemia. This is due to the fact that there is a higher emphasis placed on the peripheral lymphocyte count in the CONUT score. In addition, total cholesterol concentration which is not included in the PNI is an additional factor evaluated in the CONUT score. This is why the CONUT score is considered to be able to detect the patients who will have a poor prognosis more sensitively than the PNI.

Based on the results of the present study, it is thought that the use of the preoperative CONUT score could enable the stratification of risk for poor survival and help to individualize treatments. In clinical practice, patients with a higher risk of death from colorectal cancer can be selected based on the preoperative immune-nutritional status. The administration of more effective adjuvant chemotherapy to reduce the risk of recurrence and shorten the follow-up interval in order to diagnose early recurrence can improve the prognosis.

This study is associated with some limitations. First, this study was a retrospective, single-center design with a relatively small number of patients. Further studies, including prospective studies with a larger number of patients, should be performed to confirm our findings. Second, the ROC curve for critical cut-off used a value of 3, which was associated with a low AUC, a non-significant p-value and poor sensitivity; however, the CONUT score was significantly correlated with the outcome in this study. Third, although the CONUT was revealed to be superior to the PNI in this study, this result is based on an analysis of only 27 patients with the high CONUT/high PNI. Therefore, it may be difficult to draw any final conclusions based on such a small sample size. Fourth, there are many countries in which there are large gaps in income among individuals. Income is associated with the nutritional status and medical treatment. Under the Japanese Social Security system, the entire population is guaranteed the right to a minimum standard of living and affordable medical treatment. Therefore, there are fewer problems of malnutrition and poor medical care due to poverty in Japan. Likewise, chemotherapy is widely available and is appropriately used to treat patients. There are no significant gaps in income between the rich and poor in Japan. On the other hand, it might be necessary to consider this situation in other countries.

## Conclusion

The results of this study suggest that the CONUT score is a strong independent predictor of survival among CRC patients. Furthermore, the CONUT score might be a more sensitive prognostic factor than the PNI.
